# Pitfall of endoscopic ultrasound-guided hepaticoduodenostomy: Impact of hepatic parenchymal volume during device insertion

**DOI:** 10.1055/a-2707-3324

**Published:** 2025-10-02

**Authors:** Takeshi Ogura, Jun Matsuno, Takafumi Kanadani, Junichi Nakamura, Hiroki Nishikawa

**Affiliations:** 1 38588 Endoscopy Center, Osaka Medical and Pharmaceutical University Hospital, Osaka, Japan; 2 13010 2nd Department of Internal Medicine, Osaka Medical and Pharmaceutical University, Osaka, Japan

Endoscopic ultrasound-guided hepaticoduodenostomy (EUS-HDS) can be indicated for isolated right hepatic duct obstruction after unsuccessful endoscopic retrograde cholangiopancreatography (ERCP) 1 2 . Although this technique has clinical impact on selected patients, the technical aspects are not yet established because there are few indications for EUS-HDS itself. Compared with EUS-guided hepaticogastrostomy (HGS), pushing force may be transmitted less effectively to the bile duct, and pushback can occur during device insertion. During EUS-HGS, increasing the angle of EUS can increase the pushing force. During EUS-HDS, however, increasing the angle reduces pushback less effectively. To increase the pushing force, hepatic parenchyma volume might be important as a greater volume may reinforce the pushing force. We herein describe a pitfall of EUS-HDS during stent delivery system insertion.

After inserting the echoendoscope into the duodenum, the right hepatic bile duct was identified. Superficial intrahepatic bile duct puncture was performed using a 19G needle, and contrast medium was injected ( Fig. 1 ). After successful guidewire deployment, ERCP catheter insertion was attempted but was unsuccessful because the pushing force deflected to the right side ( Fig. 2 ). Therefore, tract dilation was performed using a drill dilator ( Fig. 3 ). Attempted insertion of a stent delivery system (5.9 Fr, HANARO Benefit; Boston Scientific, Marlborough, Massachusetts) into the biliary tract failed for the same reason ( Fig. 4 ). We then performed the double-guidewire technique to fit the axis and improve pushing ability, which enabled insertion of the stent delivery system and subsequent stent deployment ( Fig. 5 , Video 1 ). As free air was observed after the procedure, the patient was placed under conservative treatment for four days.

**Fig. 1:**
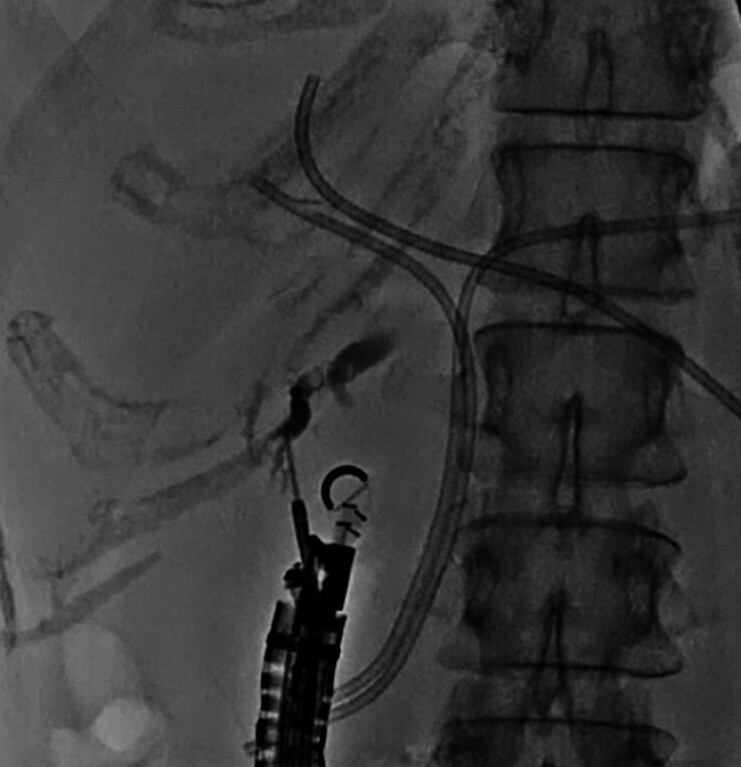
Cholangiography confirms the superficial right hepatic duct puncture.

**Fig. 2:**
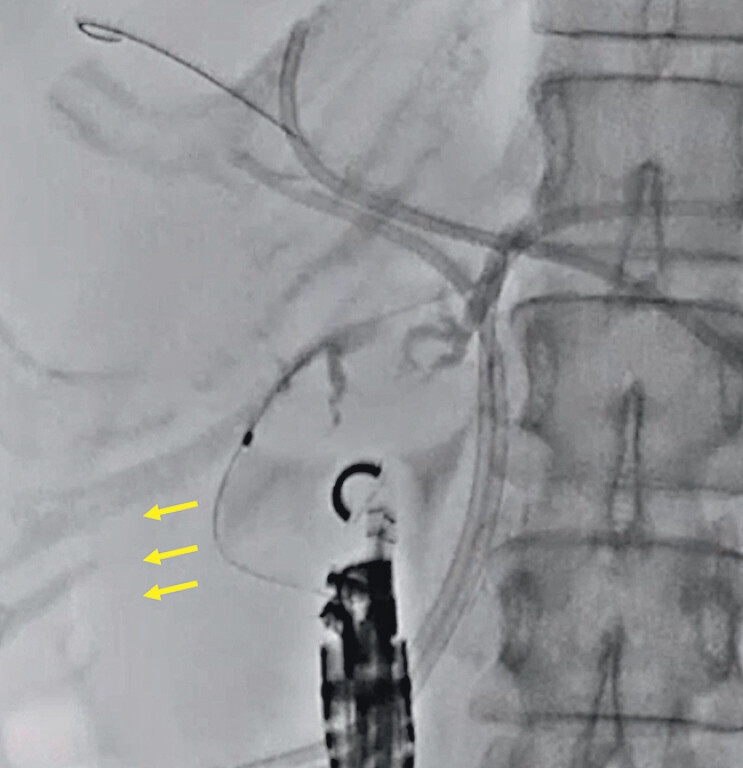
During endoscopic retrograde cholangiopancreatography catheter insertion, the pushing force deflects to the right side (arrow).

**Fig. 3:**
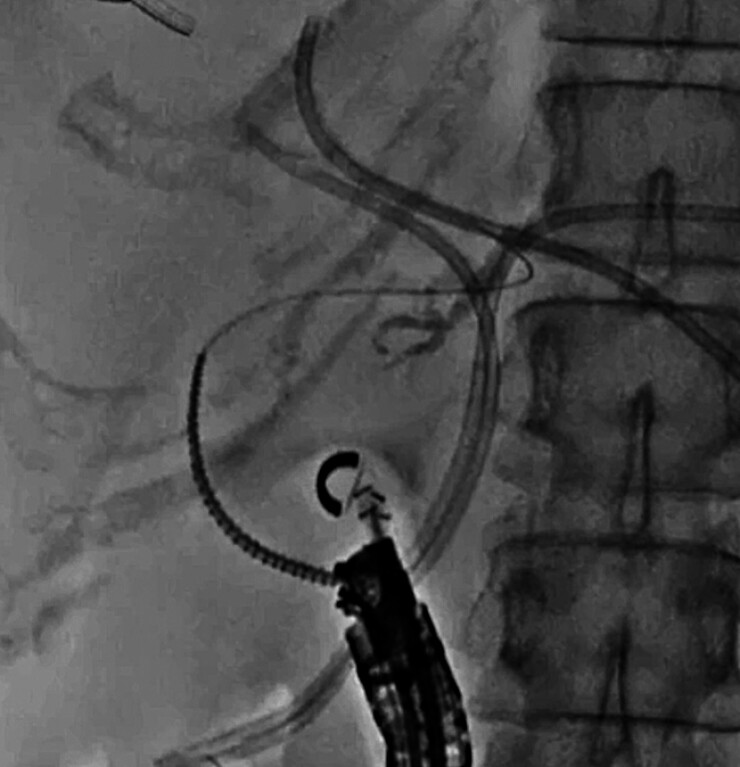
Tract dilation is performed using the drill dilator.

**Fig. 4:**
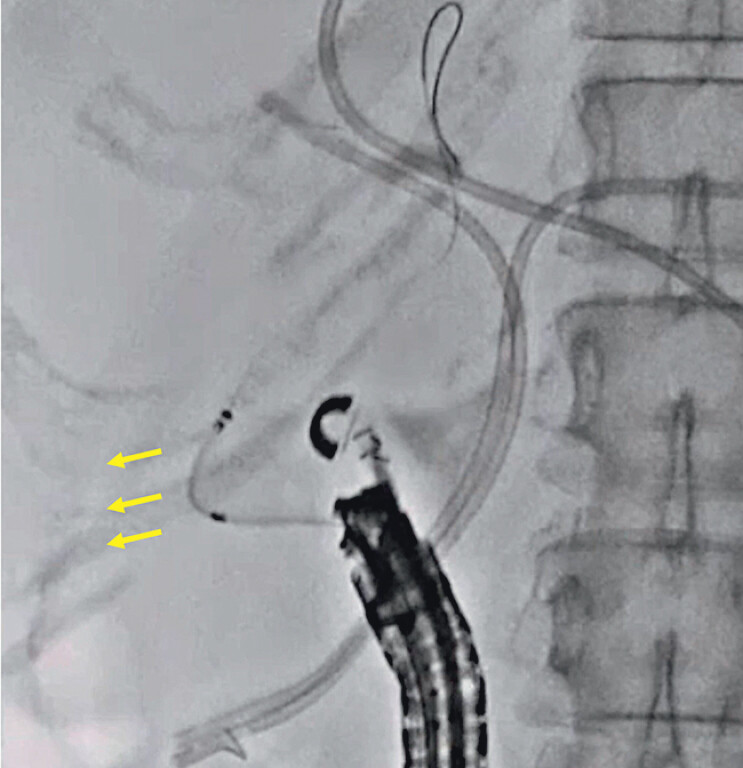
Insertion of the stent delivery system failed due to the pushing force deflecting to the right side (arrow).

**Fig. 5:**
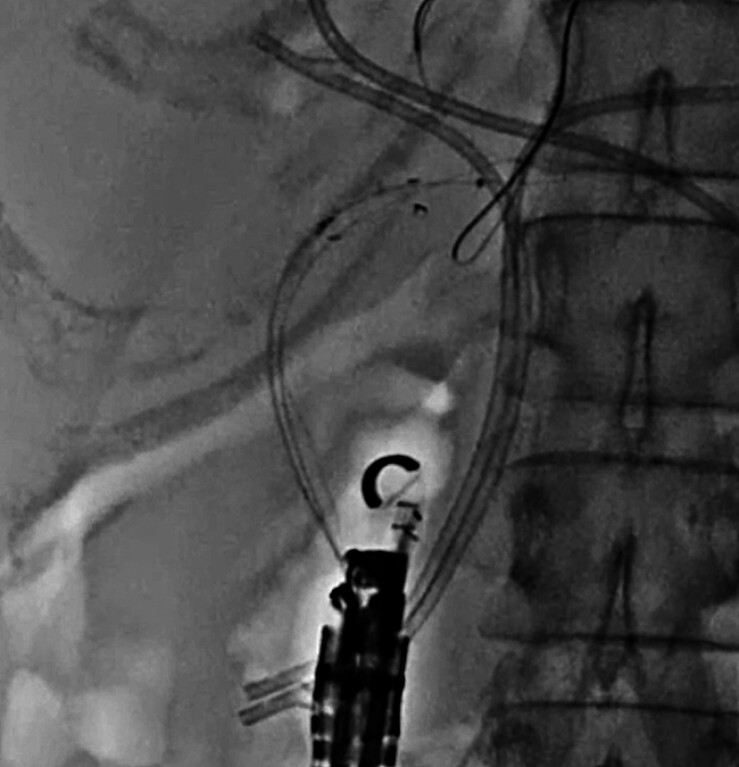
Successful stent deployment after employing the double-guidewire technique.

Insertion of the stent delivery system failed due to the pushing force deflecting to the right side.Video 1

In conclusion, choosing a puncture route across a large enough hepatic parenchyma volume may enable successful device insertion during EUS-HDS.

Endoscopy_UCTN_Code_TTT_1AS_2AD

## Notes

Endoscopy E-Videos https://eref.thieme.de/e-videos
*E-Videos* is an open access online section of the journal *Endoscopy* , reporting on interesting cases and new techniques in gastroenterological endoscopy. All papers include a high-quality video and are published with a Creative Commons CC-BY license. Endoscopy E-Videos qualify for HINARI discounts and waivers and eligibility is automatically checked during the submission process. We grant 100% waivers to articles whose corresponding authors are based in Group A countries and 50% waivers to those who are based in Group B countries as classified by Research4Life (see: https://www.research4life.org/access/eligibility/ ). This section has its own submission website at https://mc.manuscriptcentral.com/e-videos .
